# Heparin-Induced Thrombocytopenia at the Emergency Department Due to Intermittent Heparin Flush in a Patient Undergoing Stem Cell Transplant

**DOI:** 10.7759/cureus.31798

**Published:** 2022-11-22

**Authors:** Kyaw Z Thein, Sarah A Elsaim, Maggie Q Ma, Cristhiam M Rojas Hernandez, Ahmed Elsayem

**Affiliations:** 1 Target Therapy, University of Texas MD (Monroe Dunaway) Anderson Cancer Center, Houston, USA; 2 Medical School, University of Texas MD (Monroe Dunaway) Anderson Cancer Center, Houston, USA; 3 Clinical Pharmacy, University of Texas MD (Monroe Dunaway) Anderson Cancer Center, Houston, USA; 4 Hematology, University of Texas MD (Monroe Dunaway) Anderson Cancer Center, Houston, USA; 5 Emergency Medicine, University of Texas MD (Monroe Dunaway) Anderson Cancer Center, Houston, USA

**Keywords:** cancer, emergency, argatroban, adverse event, thrombocytopenia, heparin

## Abstract

Heparin-induced thrombocytopenia (HIT) is an adverse reaction to heparin products, but not warfarin. HIT usually occurs 5‒10 days after exposure to heparin. Here, we report a case of HIT with multiple thrombotic events and severe thrombocytopenia resulting from intermittent intravenous heparin flushes for maintenance of a newly placed subclavian central venous catheter (CVC) for stem cell transplant. The patient is a woman in her forties with multiple myeloma who presented to the emergency department (ED) with dyspnea, pleuritic-type chest pain, hemoptysis, and worsening left-leg swelling. Heparin had been used to flush the CVC. Her platelet count began dropping approximately one week after insertion. The patient was receiving other medications known to cause thrombocytopenia. She had undergone multiple platelet transfusions. In the ED, her lab results showed thrombocytopenia), anemia; renal insufficiency; and elevated troponin, prothrombin time, and D-dimer levels. Because of the hemoptysis and thrombocytopenia, she initially received platelet transfusion and oxygen. She was found to have deep vein thrombosis of the lower extremity and started a referral to interventional radiology for inferior vena cava (IVC) filter placement. However, further review and consultation of the Benign Hematology service, discussion about the timing of decreased platelet count shortly after CVC placement and heparin administration, and the presence of thrombosis, suggested a high pre-test probability of HIT. Anticoagulation with argatroban was initiated, and IVC filter insertion was canceled. Further workup confirmed HIT diagnosis and saddle pulmonary embolism. During the patient's hospitalization, her platelets continued to improve and reached baseline upon discharge. She was transitioned to fondaparinux at the time of discharge. A few weeks later, she had successful stem cell transplantation. Emergency physicians treating patients with thrombocytopenia receiving heparin, even in small amounts, should consider the possibility of HIT and be familiar with its management.

## Introduction

Heparin-induced thrombocytopenia (HIT) type II is a clinicopathologic syndrome as a result of an immune-mediated adverse reaction to heparin products [1,2]. Several tools are useful for predicting the probability of HIT, the most widely validated of which are the 4Ts (Thrombocytopenia, Timing (onset of platelet count falling 5 -10 days from heparin exposure), Thrombosis, and absence of other causes of Thrombocytopenia) score [2,3]. Retrospective data from our institution has demonstrated that the currently available clinical prediction tools for HIT may perform accurately in the cancer population [3]. Nonetheless, HIT remains a diagnostic challenge, particularly when other causes of thrombocytopenia are present. Although the intravenous continuous infusion of unfractionated heparin is well known to cause HIT, the effect of intermittent heparin flushes is commonly overlooked in the differential diagnosis. Here, we report a case of HIT with multifocal venous thrombotic events and severe thrombocytopenia resulting in bleeding caused by intermittent heparin flushes. Heparin was given to maintain a newly placed subclavian central venous catheter (CVC) in a stem cell transplant candidate who presented to our emergency department (ED).

## Case presentation

A woman in her mid-forties came to the ED with dyspnea, pleuritic-type chest pain, cough with hemoptysis, and worsening swelling in her left leg. The patient had multiple myeloma treated with chemotherapy and was planning to undergo stem cell transplantation. Stem cell mobilization had been initiated with filgrastim and plerixafor. Three weeks prior to the ED presentation, a CVC was inserted, and heparin was used to flush the line daily. Her platelet count started to drop approximately seven days after insertion, decreasing from around 200,000 cells/µL to as low as 5,000 cells/µL; as a result, she had undergone multiple platelet transfusions. Table 1 shows platelet changes and major interventions since inferior vena cava (IVC) catheter placement and the start of the heparin flush. Of note, she was on other medications that could have caused thrombocytopenia, including recently administered chemotherapy with lenalidomide and bortezomib, and other medications with a lower likelihood of causing thrombocytopenia: omeprazole, bumetanide, spironolactone, montelukast, acyclovir, and clonazepam.

**Table 1:** Clinical course and platelet counts during the observed period GCSF: granulocyte colony-stimulating factor; ED: emergency department; LLE: left lower extremity; DVT: deep vein thrombosis; Hit: heparin-induced thrombocytopenia; RUE: right upper extremity; RLE: right lower extremity

Day	Platelet count (× 10^3^ cells/µL)	Remarks
1	200	Baseline platelet count. Right side port-a-cath with intermittent heparin flushes
5	163	Begin apheresis with GCSF and plerixafor
7	69	Continue apheresis with GCSF and plerixafor
8-20	5 to 33	Collected 5.77 x 10^6^ stem cells, received multiple platelet transfusions, transplant was delayed because of slow recovery of platelets
22	9	Presented to ED with shortness of breath, hemoptysis, LLE DVT, received platelet transfusion in ED
23	32	Started on argatroban infusion, HIT antibody came back positive with optic density of 2.997, serotonin release assay was ordered
24	15	Saddle pulmonary embolism, hemorrhage, or infarction in the left lower lobe, worsening left lower extremity edema with signs of phlegmasia cerulea dolens. Vascular surgery is recommended if the patient is not a good candidate for thrombolysis due to thrombocytopenia.
25	14	Positive for DVT in RUE, RLE
32	66	Serotonin release assay came back positive
36	141	Transitioned to fondaparinux
39	194	Discharge on fondaparinux, had transplant on 10/22/15, and completed six months of fondaparinux without complication

In the ED, the patient’s vital signs were remarkable for a heart rate of 96 and oxygen saturation of 90% on room air. She was placed on 2 liters of oxygen by nasal cannula. The patient expressed symptoms of shortness of breath and anxiety. Her physical examination was remarkable for bilateral lower-extremity edema, mild petechia, and some anxiety. Labs on presentation to the ED were significant for a platelet count of 9,000 cells/µL (as compared to 200,000 cells/µL on the day of CVC insertion). Other laboratory values included serum creatinine of 1.8 mg/dL, troponin I of 0.12 ng/mL, D-dimer >20 mcg/mL, prothrombin time of 16.9 seconds, and a white blood count of 15,600 cells/µL. Electrocardiography showed a right bundle branch block with right axis deviation, and the chest X-ray was unremarkable. A Doppler ultrasound of the lower extremity revealed an extensive thrombosis in the left external iliac to the left popliteal vein. Bedside echocardiography revealed left ventricular dilatation but no right ventricular strain. Although pulmonary embolism was highly suspected, computed tomography imaging with contrast could not be completed due to acute kidney injury. Because of hemoptysis and thrombocytopenia, platelets were transfused and interventional radiology service was consulted for IVC filter placement. However, after careful deliberation, we began to suspect HIT because of the decreasing platelet count in concert with the timing of CVC placement and administration of heparin, the severity of thrombocytopenia, and presence of thrombosis. Her 4T score was 7 (two points for thrombocytopenia, two points for timing, two points for thrombosis, and one point for the absence of other causes of thrombocytopenia), correlating with a high probability of HIT. We gave one point rather than two for the last “T” because the patient could have other causes for thrombocytopenia. Hematology service was consulted and argatroban infusion was subsequently initiated. The IVC filter insertion was canceled, and platelet transfusion was curtailed. The patient was admitted to the hospital, where further workup revealed multiple deep vein thromboses, including in the right upper extremity, and a saddle pulmonary embolism. Enzyme-linked immunosorbent assay (ELISA) for HIT antibody (IgG PF4 optical density of 2.879) and serotonin-release assays were positive. The patient’s platelet count continued to improve on argatroban, reaching 194,000/µL by the time she was discharged (Table 1). She was eventually transitioned to fondaparinux (after her creatinine returned to baseline). She underwent stem cell transplant five weeks later and completed a six-month course of fondaparinux without complications.

## Discussion

HIT is a paradoxical reaction of venous and arterial thrombosis when heparin-dependent platelet-activating IgG antibodies bind to platelet factor 4/heparin complexes on the platelet surface, resulting in thrombocytopenia and activation of platelets leading to platelet aggregation and thrombosis [4, 5]. Isolated HIT increases the risk of thrombosis by up to 50% and increases mortality by 10‒30% [6]. HIT usually occurs 5‒10 days after exposure to unfractionated heparin, low-molecular-weight heparin, or, rarely, fondaparinux [7]. HIT is an uncommon clinicopathological syndrome; the incidence varies in different studies and mostly ranges from 0.1% to 5.0%, depending on individual risk factors and type and amount of heparin exposure [8,9]. In the case of subcutaneous prophylactic heparin exposure or lower doses of heparin exposure, including the very small amounts introduced through heparin-coated catheters, the incidence of HIT can be 0.5‒1.0% [10-13]. Our case may be applicable to other intermittent heparin exposures such as in dialysis patients or in cardiac surgeries. Alternative anticoagulation with non-heparin products such as argatroban, bivalirudin, or danaparoid is the primary way that HIT is managed [14-16]. The few reported cases of fondaparinux-related HIT were successfully managed [16-18].

In our patient, suspicion of HIT was missed initially because her drop in platelet count was masked by apheresis or immobilization with granulocyte-colony stimulating factor and plerixafor, which also causes thrombocytopenia. Thrombosis risk in HIT is correlated with the severity of thrombocytopenia and lower platelet counts, which lead to platelet transfusions that can contribute to additional thrombosis [19]. As the presentation of HIT was obscured in our patient, IVC filter placement was initially proposed, yet this could have been detrimental in the setting of HIT [20]. The patient did not develop any complications or bleeding from the use of fondaparinux and underwent a stem cell transplant successfully while on fondaparinux. With multiple comorbidities in cancer population and different procedures and medications given, ED physicians and other clinicians should have a high Index of suspicion for HIT and be familiar with its management.

## Conclusions

The presence of acute thrombosis in a patient with new thrombocytopenia merits considering the possibility of HIT, including any recent history of exposure to heparin. ED physicians, and interventional radiologists, should evaluate for HIT before placing IVC filter in a patient with acute thrombosis and thrombocytopenia; since the optimal management of HIT will require therapeutic anticoagulation, regardless of the platelet count. With multiple comorbidities in cancer population and different procedures and medications given, clinicians should have high Index of suspicion for HIT and be familiar with its management.

## References

[1] Linkins LA (2015). Heparin induced thrombocytopenia. BMJ.

[2] Linkins LA, Bates SM, Lee AY, Heddle NM, Wang G, Warkentin TE (2015). Combination of 4Ts score and PF4/H-PaGIA for diagnosis and management of heparin-induced thrombocytopenia: prospective cohort study. Blood.

[3] Wong M, Oo TH, Qiao W, Garg N, Rojas-Hernandez CM (2017). Performance of 4T score and heparin-platelet factor 4 antibody in the diagnosis of heparin-induced thrombocytopenia (HIT) in cancer. J Thromb Thrombolysis.

[4] Warkentin TE, Levine MN, Hirsh J, Horsewood P, Roberts RS, Gent M, Kelton JG (1995). Heparin-induced thrombocytopenia in patients treated with low-molecular-weight heparin or unfractionated heparin. N Engl J Med.

[5] Warkentin TE, Kelton JG (2001). Delayed-onset heparin-induced thrombocytopenia and thrombosis. Ann Intern Med.

[6] Warkentin TE, JG Kelton (1996). A 14-year study of heparin-induced thrombocytopenia. Am J Med.

[7] Warkentin TE, Kelton JG (2001). Temporal aspects of heparin-induced thrombocytopenia. N Engl J Med.

[8] Warkentin TE, Sheppard JA, Moore JC, Moore KM, Sigouin CS, Kelton JG (2005). Laboratory testing for the antibodies that cause heparin-induced thrombocytopenia: how much class do we need?. J Lab Clin Med.

[9] Lindhoff-Last E, Nakov R, Misselwitz F, Breddin HK, Bauersachs R (2002). Incidence and clinical relevance of heparin-induced antibodies in patients with deep vein thrombosis treated with unfractionated or low-molecular-weight heparin. Br J Haematol.

[10] Kadidal VV, Mayo DJ, Horne MK (1999). Heparin-induced thrombocytopenia (HIT) due to heparin flushes: a report of three cases. J Intern Med.

[11] Heeger PS, Backstrom JT (1986). Heparin flushes and thrombocytopenia. Ann Intern Med.

[12] Ling E, Warkentin TE (1998). Intraoperative heparin flushes and subsequent acute heparin-induced thrombocytopenia. Anesthesiology.

[13] Laster J, Silver D (1988). Heparin-coated catheters and heparin-induced thrombocytopenia. J Vasc Surg.

[14] Lewis BE, Wallis DE, Berkowitz SD (2001). Argatroban anticoagulant therapy in patients with heparin-induced thrombocytopenia. Circulation.

[15] Hirsh J, Heddle N, Kelton JG (2004). Treatment of heparin-induced thrombocytopenia: a critical review. Arch Intern Med.

[16] Warkentin TE, Greinacher A, Koster A, Lincoff AM (2008). Treatment and prevention of heparin-induced thrombocytopenia: American College of Chest Physicians Evidence-Based Clinical Practice Guidelines (8th Edition). Chest.

[17] Kang M, Alahmadi M, Sawh S, Kovacs MJ, Lazo-Langner A (2015). Fondaparinux for the treatment of suspected heparin-induced thrombocytopenia: a propensity score-matched study. Blood.

[18] Efird LE, Kockler DR (2006). Fondaparinux for thromboembolic treatment and prophylaxis of heparin-induced thrombocytopenia. Ann Pharmacother.

[19] Goel R, Ness PM, Takemoto CM, Krishnamurti L, King KE, Tobian AA (2015). Platelet transfusions in platelet consumptive disorders are associated with arterial thrombosis and in-hospital mortality. Blood.

[20] Ishibashi H, Takashi O, Hoska M (2005). Heparin-induced thrombocytopenia complicated with massive thrombosis of the inferior vena cava after filter placement. Int Angiol.

